# Role of β-Catenin in Post-Meiotic Male Germ Cell Differentiation

**DOI:** 10.1371/journal.pone.0028039

**Published:** 2011-11-18

**Authors:** Yao-Fu Chang, Jennifer S. Lee-Chang, Krystle Y. Harris, Amiya P. Sinha-Hikim, Manjeet K. Rao

**Affiliations:** 1 Greehey Children's Cancer Research Institute, The University of Texas Health Science Center at San Antonio, San Antonio, Texas, United States of America; 2 Department of Cellular and Structural Biology, The University of Texas Health Science Center at San Antonio, San Antonio, Texas, United States of America; 3 Division of Endocrinology, Metabolism, and Molecular Medicine, Charles R. Drew University of Medicine and Science, Los Angeles, California, United States of America; Northwestern University Feinberg School of Medicine, United States of America

## Abstract

Though roles of β-catenin signaling during testis development have been well established, relatively little is known about its role in postnatal testicular physiology. Even less is known about its role in post-meiotic germ cell development and differentiation. Here, we report that β-catenin is highly expressed in post-meiotic germ cells and plays an important role during spermiogenesis in mice. Spermatid-specific deletion of β-catenin resulted in significantly reduced sperm count, increased germ cell apoptosis and impaired fertility. In addition, ultrastructural studies show that the loss of β-catenin in post-meiotic germ cells led to acrosomal defects, anomalous release of immature spermatids and disruption of adherens junctions between Sertoli cells and elongating spermatids (apical ectoplasmic specialization; ES). These defects are likely due to altered expression of several genes reportedly involved in Sertoli cell-germ cell adhesion and germ cell differentiation, as revealed by gene expression analysis. Taken together, our results suggest that β-catenin is an important molecular link that integrates Sertoli cell-germ cell adhesion with the signaling events essential for post-meiotic germ cell development and maturation. Since β-catenin is also highly expressed in the Sertoli cells, we propose that binding of germ cell β-catenin complex to β-catenin complex on Sertoli cell at the apical ES surface triggers a signaling cascade that regulates post-meiotic germ cell differentiation.

## Introduction

β-catenin is highly expressed in fetal Sertoli cells and the germ cells of mice. Recent studies have shown that perturbation of β-catenin signaling in embryonic Sertoli cells results in testicular degeneration, testicular cord disruption, and Mullerian duct regression [1], [2], [3]. Similarly, aberrant activation of β-catenin leads to impaired development of primordial germ cells [4]. β-catenin expression also persists in Sertoli and germ cells of the adult testis [5], [6]. In particular, β-catenin is found in the ectoplasmic specialization (ES), a testis-specific adherens junction formed between Sertoli cells at the basal compartment (basal ES), site of the blood-testis barrier, as well as between Sertoli/germ cells at the adluminal compartment (apical ES) of the seminiferous epithelium [7]. Despite being an integral unit of the ES, which is critical for germ cell differentiation and maturation, the role of β-catenin in adult germ cells is not clearly documented. Even less is known about the expression and function of β-catenin in post-meiotic germ cells. Since the β-catenin-cadherin complex is essential for adherens junction formation and stability as well as cell-cell signaling in epithelial cells [8], we reasoned that β-catenin may play an important role in germ cell maturation by regulating adhesion and signaling events at the Sertoli cell-germ cell interface.

To address β-catenin's role during germ cell differentiation, we deleted β-catenin specifically in haploid spermatids. Inactivation of β-catenin in post-meiotic germ cells resulted in increased germ cell apoptosis, compromised sperm motility, acrosomal defects, abnormal chromatin compaction, and loss of Sertoli cell-germ cell adhesion at the apical ES, leading to impaired fertility. These defects may be due to altered levels of several genes associated with cell-cell signaling and cell adhesion in β-catenin-deleted germ cells. Further supporting the notion that β-catenin may be a critical regulator of Sertoli cell-germ cell adhesion were our findings that β-catenin expression was localized to the distal portion of spermatids (the side normally in close contact with Sertoli cells [9]) and that β-catenin associated with JAM-C, a protein known to be crucial for Sertoli cell-/post-meiotic germ cell-adhesion [10]. Deletion of β-catenin also resulted in the dysregulation of an actin-associated protein Arpc5 that we have recently identified to be a translational suppressor, which regulates chromatin compaction in post-meiotic germ cells. Taken together, our results suggest that β-catenin expression in spermatids regulates specific events necessary for proper differentiation and maturation of post-meiotic germ cells.

## Results

### β-catenin expression in post-meiotic germ cells

**Figure 1:**
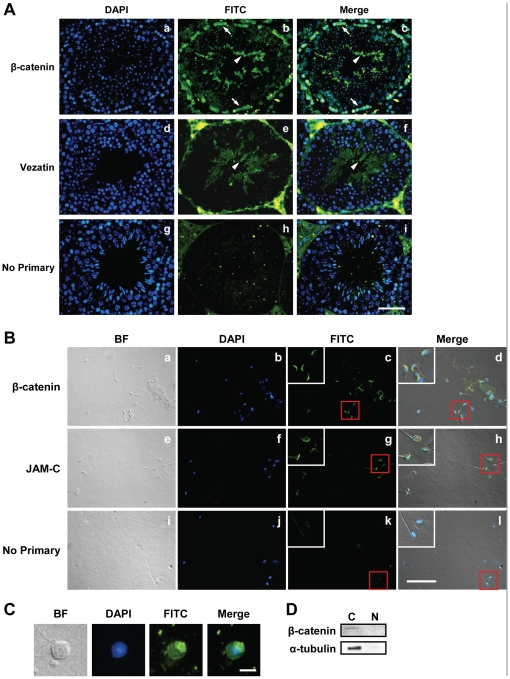
β-catenin is highly expressed in post-meiotic germ cells. (**A**) β-catenin is expressed at the apical ectoplasmic specializations (ES) and basal compartment in testis seminiferous tubules. Testis sections were labeled with anti-β-catenin (1∶200; Sigma; panels a-c) or anti-vezatin (a protein reported to be expressed only at the apical ES [76]; 1∶200; Santa Cruz; panels d-f), followed by FITC-conjugated goat anti-rabbit secondary antibody (1∶150; Zymed). Sections were counterstained with DAPI (blue) for nuclear staining. Arrowheads indicate the apical ES, and arrows indicate the Sertoli cell/germ cell cytoplasm at the basal compartment. Scale bar, 50 µm. (**B**) β-catenin localization in elongated spermatids. Purified spermatids were labeled with anti-β-catenin (1∶50; Upstate; panels a-d) or anti-JAM-C (1∶50; Santa Cruz; panels e-h). Secondary antibodies were FITC-conjugated goat anti-rabbit (1∶100) for β-catenin and FITC-conjugated goat anti-rat (1∶100; Zymed) for JAM-C. Spermatids were counterstained with DAPI (blue) for nuclear staining. Areas in red boxes are magnified in insets. Scale bar, 50 µm. (**C**) Immunofluorescence staining of β-catenin in late round spermatids. Slides were treated as described in (**B**). Scale bar, 10 µm. (**D**) β-catenin is localized to the cytoplasm in the testis. Western blot of germ cell nuclear and cytoplasmic fractions using anti-β-catenin antibody (1∶3000; Sigma). The blot was stripped and reprobed with anti-α-tubulin (1∶2000; Sigma) to show the purity of the fractions.

The expression of β-catenin in Sertoli cell of the postnatal mouse testis is well documented [6]; however, its expression in germ cells, particularly in post-meiotic germ cells, is not clear. To determine the expression pattern of β-catenin in testicular germ cells, we enriched pre- and post-meiotic germ cell by centrifugal elutriation as described previously [11]. Quantitative real-time RT-PCR (qPCR) analysis on mRNA from enriched testicular cell populations showed high levels of β-catenin expression in Sertoli cells as well as different germ cell populations (including round and elongating spermatids), when compared with known Sertoli cell, pre-meiotic germ cell, and post-meiotic germ cell-specific markers (Table S1). To further substantiate these findings, we performed immunofluorescence studies on seminiferous tubule sections. Consistent with our qPCR results, β-catenin was found to be highly expressed in both basal (pre-meiotic germ cells) and apical (post-meiotic germ cells) compartments of the seminiferous epithelium (Figure 1A). Sub-cellular localization studies on enriched spermatogenic cell populations revealed that β-catenin expression in late round spermatids and elongating/elongated spermatids was confined primarily to the apical and distal side of the head, respectively, the sides which are normally in close contacts with Sertoli cells (Figure 1B, panels a-d, and 1C). The localization of β-catenin in spermatid head is similar to the expression pattern of JAM-C (Figure 1B, panels e-h), a protein highly expressed in spermatids and the loss of which causes disruption of Sertoli-spermatid adhesion resulting in impaired germ cell differentiation [10]. Next, we determined the sub-cellular distribution of β-catenin in enriched germ cells. As shown in Figure 1D, β-catenin expression was predominantly cytoplasmic.

### β-catenin deletion results in impaired post-meiotic germ cell development

**Figure 2:**
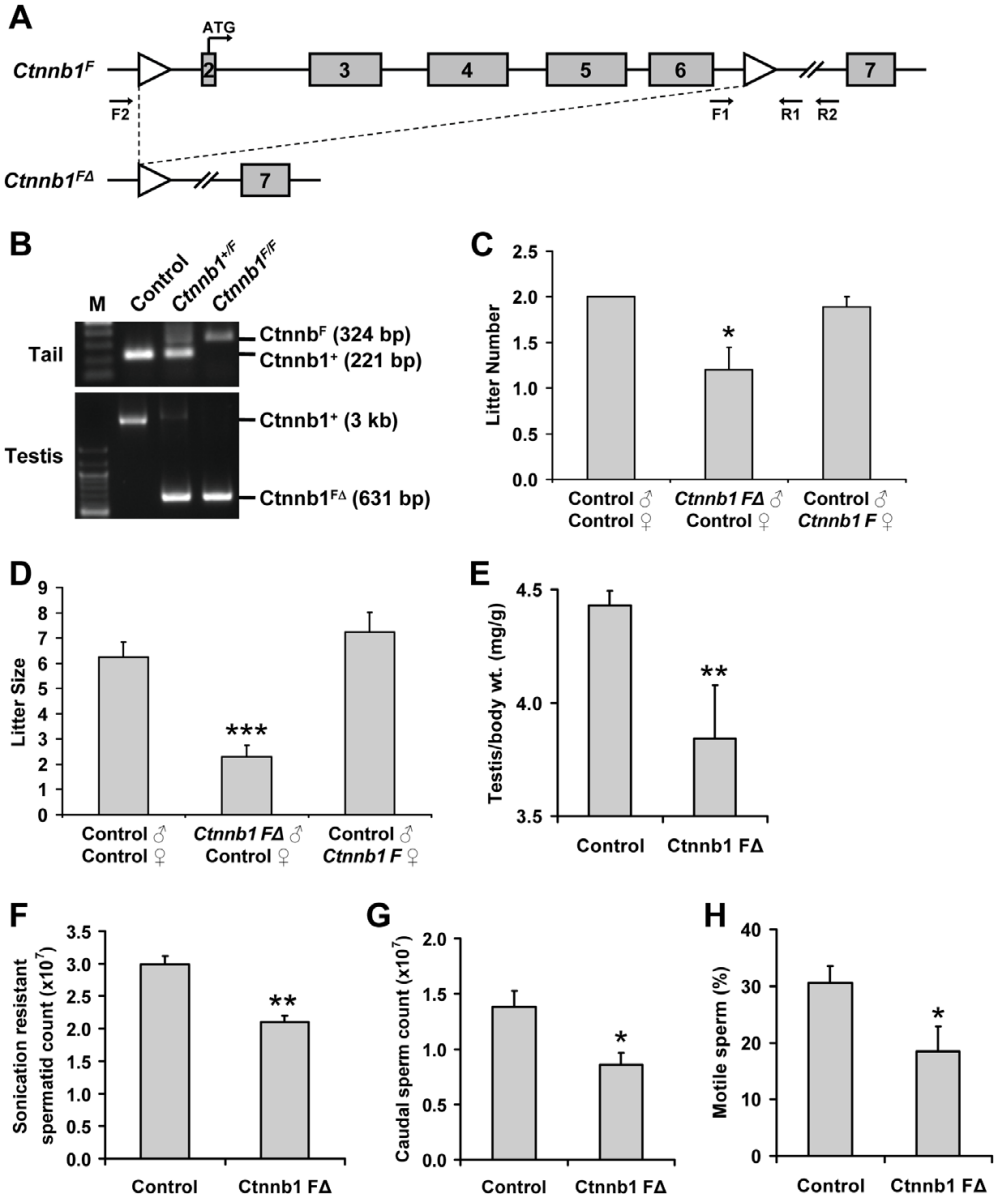
Post-meiotic germ cell-specific inactivation of β-catenin and associated reproductive defects. (**A**) Schematic of the *β-catenin-flox* allele before (*Ctnnb1^F^*) and after (*Ctnnb1^FΔ^*) *Prm1-cre*-mediated recombination. β-catenin exons are numbered. F1, F2, R1, and R2 represent primers used for genotyping. (**B**) PCR genotype analyses of tail or testis genomic DNA using primers F1 and R1 or F2 and R2, respectively. Sequences and product sizes have been previously described [12]. (**C**) and (**D**) *Prm1-cre* hemizygous*-*β*-catenin-flox* homozygous (*Ctnnb1 F*Δ) male mice are severely sub-fertile. (**C**) Mean number of litters (*n* = 10, *p<0.05) and (**D**) mean number of pups per litter (*n* = 10, ***p<0.0001) obtained from eight-week timed matings of 6 to 8-week old *Ctnnb1 F*Δ mice and control littermates. (**E**) *Ctnnb1 F*Δ male mice exhibited significantly reduced testis to body weight ratio (*n* = 9, *p<0.05). (**F**) *Ctnnb1 F*Δ mice had significantly lower sonication-resistant spermatids count (*n* = 3, **p<0.01). (**G**) Caudal epididymal sperm count showed significantly fewer sperm in *Ctnnb1 F*Δ mice (*n* = 9, *p<0.05). (**H**) Significantly reduced number of caudal epididymal sperm with forward motility in *Ctnnb1 F*Δ mice (*n* = 9, *p<0.05).

To address the role of β-catenin in post-meiotic germ cell development, we generated β-catenin conditional knockout mice by mating transgenic mice expressing *Prm1*- promoter-driven cre recombinase with β-catenin floxed mice (Figure 2A and 2B), which have been described previously [12], [13]. Since expression of the cre transgene is driven by *Prm1* promoter, which we and others have shown to be active only in round spermatids (as early as postnatal day 18; Chang *et al.*, unpublished observations, in submission; [14]), we expected that recombination would be restricted to post-meiotic germ cells. Indeed, loss of β-catenin expression was observed only in haploid spermatids, but not in the Sertoli cells of *β-catenin*-deleted mice (Figure S1 and S2). Consistent with previous reports of highly efficient *Prm1-cre*-mediated recombination of lox sequence [13], PCR analyses of genomic DNA from pups produced from matings between *Prm1-cre* hemizygous*-* *β-catenin-flox* homozygous (labeled as *Ctnnb1 F*Δ) males and wild-type control (labeled as Control; C57BL/6×129S1/SvIm mixed background) females revealed more than 90% of recombination of the *β-catenin-flox* allele in the testis (data not shown). To determine whether β-catenin deletion in post-meiotic germ cells produced any reproductive defects, we performed fertility analyses of *Ctnnb1 F*Δ animals. Eight-week timed mating studies revealed impaired fertility of *Ctnnb1 F*Δ males, as matings between *Ctnnb1 F*Δ males and control females not only resulted in significantly fewer litters (Control ♂×Control ♀ = 2.0±0, *Ctnnb1 F*Δ ♂×Control ♀ = 1.2±0.2, Control ♂×*Ctnnb1 F* ♀ = 1.9±0.1, *n* = 10, *p<0.05; Figure 2C) but also produced fewer pups per litter (Control ♂×Control ♀ = 6.3±0.6, *Ctnnb1 F*Δ ♂×Control ♀ = 2.3±0.4, Control ♂×*Ctnnb1 F* ♀ = 7.2±0.8, *n* = 10, ***p<0.0001; Figure 2D). This reproductive defect was specific to *Ctnnb1 F*Δ males, as matings between either control males and females or control males and *Prm1-cre* hemizygous*-* *β-catenin-flox* homozygous females (*Ctnnb1 F ♀*) produced normal litter sizes and pups per litter (Figure 2C and 2D). Similarly, matings between *Prm1-cre* males and control females (or control males and *Prm1-cre* females) also produced normal litter sizes and pups per litter, suggesting that the reproductive defect did not originate from the *Prm1-cre* transgene (Fig. S3). Furthermore, the deficiency in the fertility of *Ctnnb1 F*Δ males was not due to their altered sexual behavior, as they generated a similar frequency of vaginal plugs as wild type males (data not shown).

Next, we investigated the reason for the severe sub-fertility in *Ctnnb1 F*Δ male mice. *Ctnnb1 F*Δ mice had significantly reduced testis weight and modestly reduced testis size compared to control mice (Control = 4.43±0.07 mg/g, *Ctnnb1 F*Δ = 3.84±0.24 mg/g, *n* = 9, *p<0.05; Figure 2E and Figure S4), suggesting the compromised fertility in *Ctnnb1 F*Δ mice was likely due to a testicular defect. To determine if targeted deletion of β-catenin in post-meiotic germ cells affected spermatid development, we counted sonication-resistant spermatids (the most differentiated spermatids) in *Ctnnb1 F*Δ testes. Light microscopic examination of sonicated testes revealed that *Ctnnb1 F*Δ mice possessed significantly fewer sonication-resistant spermatids than their control littermates (Control = 2.99±0.13×10^7^, *Ctnnb1 F*Δ = 2.10±0.10×10^7^, *n* = 3, **p<0.01; Figure 2F). In agreement with the impaired spermatid maturation, *Ctnnb1 F*Δ mice had significantly reduced caudal sperm count (Control = 1.38±0.15×10^7^, *Ctnnb1 F*Δ = 0.86±0.11×10^7^, *n* = 9, *p<0.05; Figure 2G) and forward motility (Control = 30.6±2.9%, *Ctnnb1 F*Δ = 18.5±4.4%, *n* = 9, *p<0.05; Figure 2H) compared to control mice.

### Increased spermatid apoptosis in *Ctnnb1 F*Δ mice

**Figure 3:**
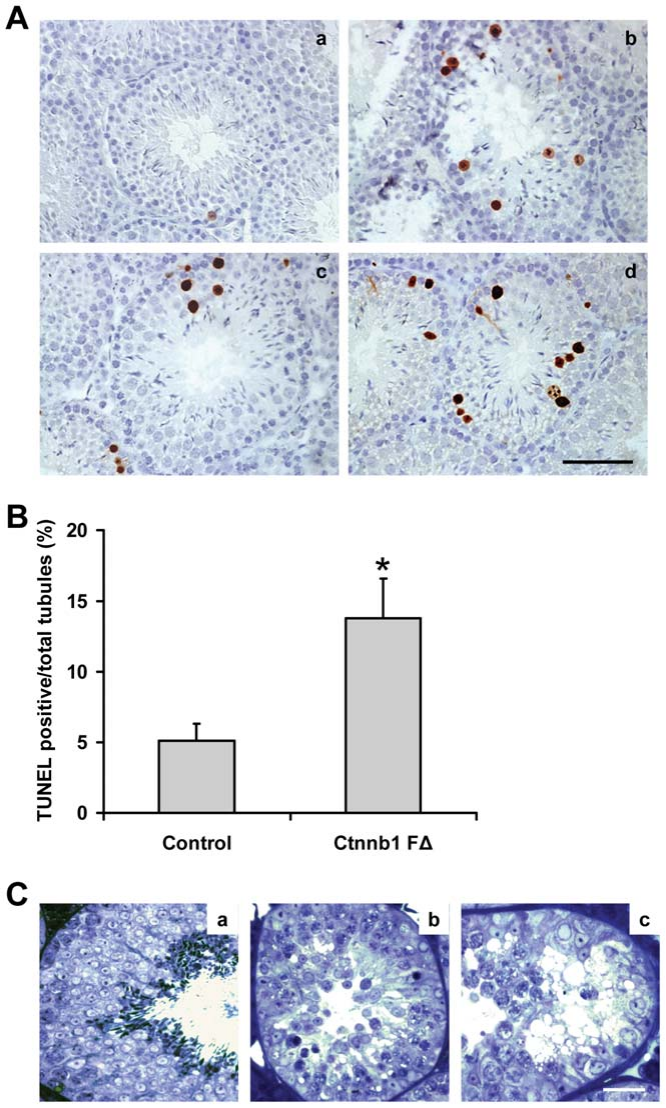
Increased germ cell apoptosis in *Ctnnb1 F*Δ mice testes. (**A**) TUNEL analysis of apoptotic germ cells in control (panel a) and *Ctnnb1 F*Δ (panels b-d) mice testes sections. Scale bar, 50 µm. (**B**) Number of TUNEL-positive cells per seminiferous tubule (*n* = 6, *p<0.05). (**C**) Light micrographs of epox-embedded and toluidine blue-stained testicular sections from control and *Ctnnb1 F*Δ mice. Tubular profiles from a control mouse testis showing Sertoli cells and germ cells at various phases of development that support normal spermatogenesis (panel a). Tubular profiles from a *Ctnnb1 F*Δ mouse showing epithelial vacuolization and complete loss of elongated spermatids (panels b and c). Scale bar, 50 µm.

We next assessed whether reduced germ cell output in *Ctnnb1 F*Δ testis was due to increased germ cell death. In normal testes, either spermatogonia or meiotic spermatocytes undergo apoptosis [15]. In agreement with this, we observed that in normal wild-type testis sections only pre-meiotic germ cells underwent apoptosis, as judged by the TUNEL assay (Figure 3A, panel a). In *Ctnnb1 F*Δ mice the number of TUNEL-positive spermatogonia and spermatocytes increased dramatically (Figure 3A, panels b-d, and 3B). This is surprising, given that the β-catenin is deleted specifically in the post-meiotic germ cells. In addition to pre-meiotic germ cells, *Ctnnb1 F*Δ mice had TUNEL-positive cells near the lumen, indicating dying round/elongating germ cells, which normally do not undergo apoptosis (Figure 3A, panel b). Consistent with this, light microscopic examination of the *Ctnnb1 F*Δ testes sections revealed that a significant number of tubules exhibited severe defects characterized by epithelial vacuolization and marked loss of elongated spermatids (Figure 3C).

### Chromatin compaction defects in *Ctnnb1 F*Δ mice

**Figure 4:**
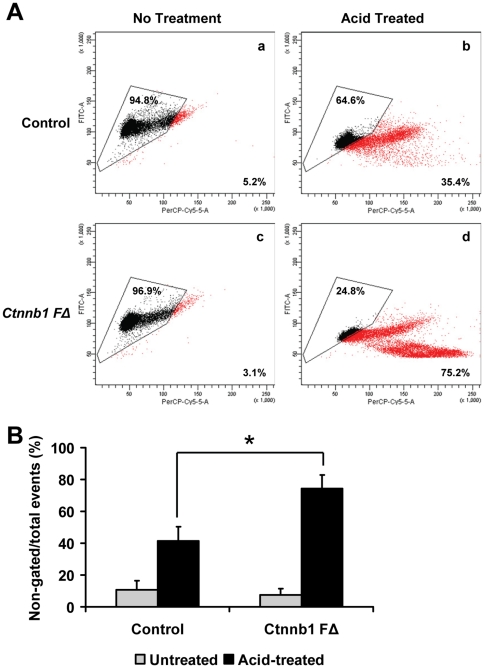
Defective chromatin compaction in *Ctnnb1 F*Δ sperm. (**A**) and (**B**) Flow cytometric analyses showing increased number of sperm with chromatin defects in *Ctnnb1 F*Δ mice. (**A**) Bivariate histograms of green versus red fluorescence for untreated and acid-denatured AO-stained sperm heads prepared by sonication from control and *Ctnnb1 F*Δ mice. Percentage inside the gated area (determined after running three control untreated samples) represents sperm with normal chromatin compaction, while percentage outside the gated area represents sperm with impaired chromatin compaction. (**B**) Percentage of non-gated/total events of acid-treated AO-stained sperm head from control and *Ctnnb1 F*Δ mice as in (**A**) (*n* = *4*, *p<0.05).

Because normal mice with significantly reduced sperm count can still maintain fertility [16], [17], we reasoned that other factors must also contribute to the severe hypofertility of *Ctnnb1 F*Δ mice. The state of sperm chromatin compaction is one important independent prognostic characteristic that is associated with fertility. Differentiating post-meiotic germ cells undergo a dynamic sequence of events resulting in condensed chromatin. Increasing evidence indicates that proper chromosomal organization in the sperm nuclei is directly correlated with fertility potential of spermatozoa [18]. To examine whether loss of β-catenin in post-meiotic germ cells may affect this process, caudal sperm from *Ctnnb1 F*Δ and control mice were acid-treated, followed by staining with acridine orange (AO). AO is a DNA intercalating dye that fluoresces green when bound to double-stranded DNA and red when bound to single-stranded DNA. As sperm with partially compacted chromatin will be more susceptible to acid denaturation, they will exhibit yellow to orange fluorescence (green plus red), and therefore can be readily distinguished from fully compacted chromatin with green fluorescence [19]. Flow cytometric analyses of AO-stained sperm heads revealed a significant increase (more than two-fold) of red fluorescence in acid-treated *Ctnnb1 F*Δ sperm (Figure 4A and 4B).

### Loss of Sertoli cell-germ cell adhesion in *Ctnnb1 F*Δ mice

**Figure 5:**
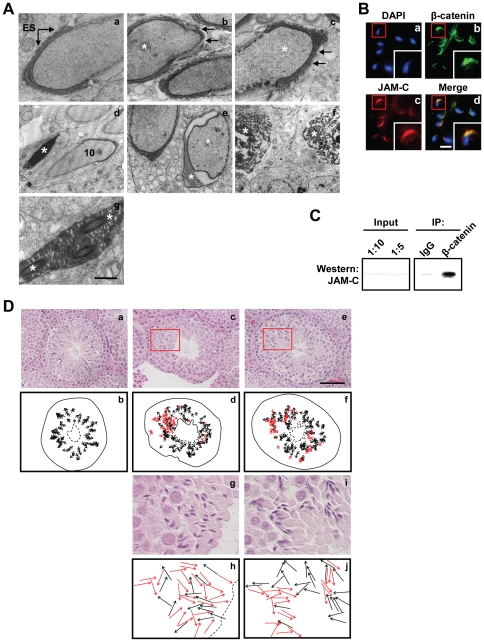
Disruption of apical ES and compromised spermatid polarity in *Ctnnb1 F*Δ mice testes. (**A**) Ultrastructural defects in *Ctnnb1 F*Δ mice. Portions of a step 10 spermatid from a control mouse shows normal morphology (panel a). The acrosome extends over the apex and over the dorsal curvature of the head. ES is well recognized and is seen over the entire acrosome region. Step 10 spermatids (asterisks) from a *Ctnnb1 F*Δ mouse show disruption and loss of ES (arrows; panels b and c). A stage X tubule from a *Ctnnb1 F*Δ mouse shows a mature spermatid that should have been released at stage VIII (asterisk; panel d). Portion of a stage XII tubule from a *Ctnnb1 F*Δ mouse shows normal and abnormal early step 10 spermatids with nuclear and acrosomal abnormalities (asterisks; panel e). *Ctnnb1 F*Δ mouse show apoptotic pachytene spermatocytes (asterisks; panel f) and mature spermatids (asterisks; panel g). Scale bar, 0.5 µm (panels a-d and g) or 1.4 µm (panels e and f). (**B**) JAM-C colocalizes with β-catenin in spermatozoa. Caudal spermatozoa were air dried on slides and fixed with 4% paraformaldehyde followed by labeling with anti-β-catenin (1∶25; Sigma; panel b) and anti-JAM-C (1∶50; panel c). Secondary antibodies were AlexaFluor 488-conjugated goat anti-rabbit (1∶400; Invitrogen) for β-catenin and AlexaFluor 594-conjugated goat anti-rat (1∶400; Invitrogen) for JAM-C. Spermatids were counterstained with DAPI (blue) for nuclear staining. Areas in red boxes are magnified in insets. Scale bar, 10 µm. (**C**) JAM-C associates with β-catenin. Co-immunoprecipitation was performed as described in Materials and Methods, and protein was visualized by western blot analyses using anti-JAM-C (1∶500). One-tenth of lysate used for co-immunoprecipitation was loaded as input. (**D**) Loss of spermatid polarity in *Ctnnb1 F*Δ testes. Testes were stained with hematoxylin and eosin (HE). Control testes sections show elongating spermatid heads pointing uniformly toward the basement membrane (black arrows; panels a-j). *Ctnnb1 F*Δ testes sections show less organization in the direction elongating spermatid heads were pointing, and contained misaligned spermatid heads pointing towards the lumen (red arrows; panels c-j). Areas in red boxes are magnified (panel c in panels g and h; panel e in panels i and j). Solid line, basement membrane; dotted line, lumen. Scale bar, 50 µm.

Since β-catenin is highly expressed in both Sertoli cells and germ cells and is reported to be an integral part of basal as well as apical ectoplasmic specialization (ES) [7], we wondered whether loss of β-catenin in spermatids had any effect on Sertoli cell-germ cell adhesion. Electron microscopic analyses revealed disruption of Sertoli cell-germ cell adhesion at the apical ES in the *Ctnnb1 F*Δ mice testes (Figure 5A, panels b-c). In contrast, seminiferous tubules from wild-type mice showed intact apical ES (Figure 5A, panel a). This is significant as apical ES is believed to play critical roles in the maturation of differentiating germ cells by controlling orientation, positioning and head morphology of the spermatid, as well as its release to the lumen [7]. Consistent with this notion, ultrastructural studies showed failure of sperm release (Figure 5A, panel d) and acrosomal defects in step 9 or 10 spermatids (Figure 5A, panel e) in *Ctnnb1 F*Δ mice testes. These defects were not observed in wild type animals. Furthermore, increased incidence of apoptosis involving pachytene spermatocytes (Figure 5A, panel f) and mature spermatids (Figure 5A, panel g) in *Ctnnb1 F*Δ mice were also evident in our ultrastructural studies.

### β-catenin and spermatid polarity

In addition to Sertoli cell-germ cell adhesion, the apical ES is believed to play important role in facilitating proper orientation of developing spermatids, such that the head of elongating/elongated spermatids point toward the basement membrane. This spermatid polarization is critical for the proper maturation of differentiating germ cells [20]. The loss of apical ES in *Ctnnb1 F*Δ mice prompted us to investigate whether β-catenin may also play a role in spermatid polarization. Since β-catenin and JAM-C, which is known to be critical for spermatid polarization [10], are both localized to the distal side of the head in elongating/elongated spermatids, we wondered if β-catenin may also play a role in spermatid polarization by interacting with JAM-C. Indeed, immunofluorescence and co-immunoprecipitation studies revealed that β-catenin interacted with JAM-C in elongating spermatids (Figure 5B and 5C). Consistent with this, many spermatids showed impaired orientation in *Ctnnb1 F*Δ testes when compared to control suggesting β-catenin to be one of the important constituent of spermatid polarity complex (Figure 5D).

### β-catenin target genes in post-meiotic germ cells

**Figure 6:**
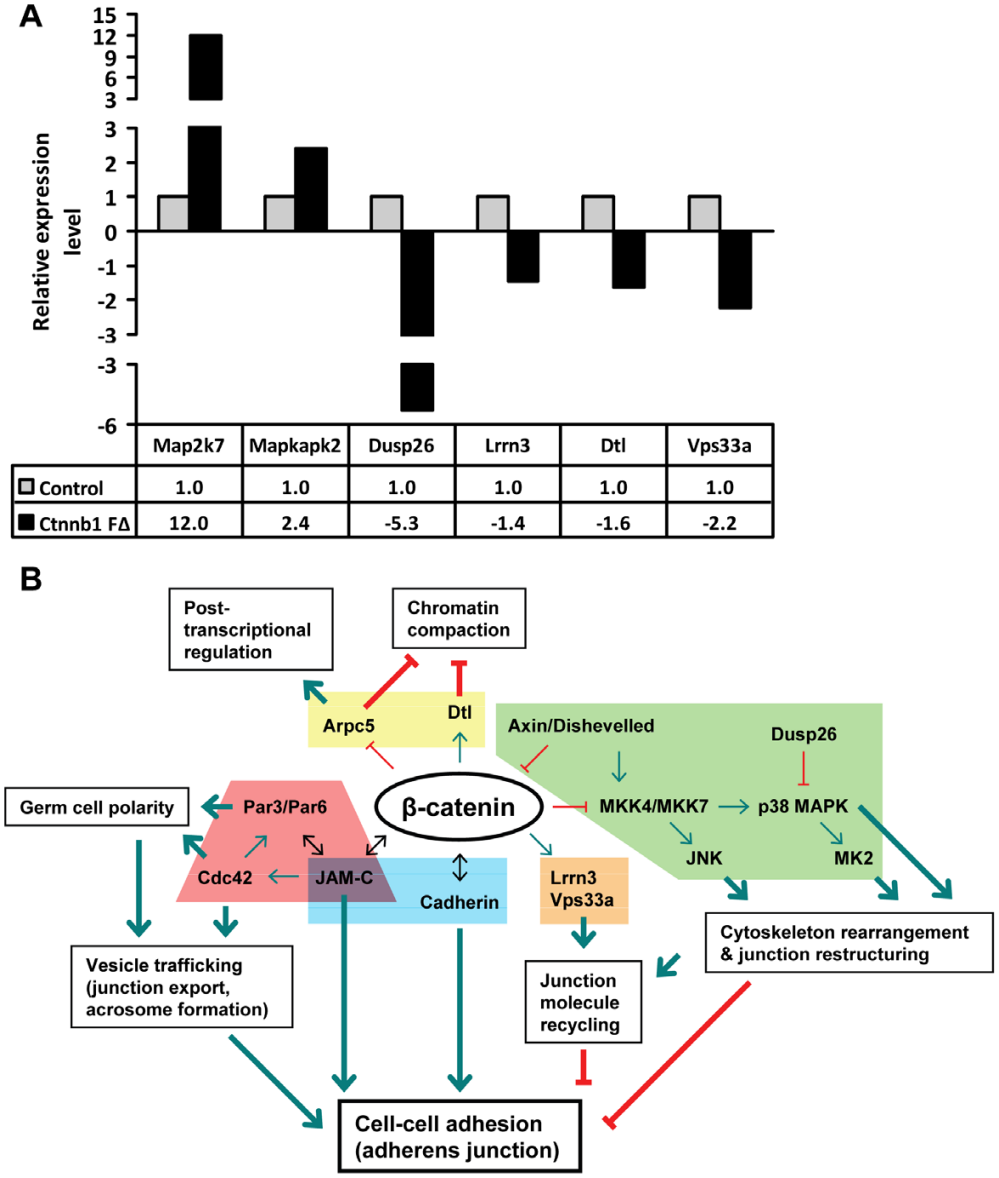
Altered gene expression in *Ctnnb1 F*Δ testes. (**A**) QPCR analyses of genes in Table S2 on purified round spermatid RNA pooled from four mice using primers in Table S3. (**B**) Model of β-catenin signaling cascade at the apical ES. Since MAPK signaling proteins play a role in actin restructuring [21], [22], [23], we propose that β-catenin may stabilize the actin cytoskeleton network on germ cell surfaces by activating negative regulators (Dusp26) and inhibiting positive regulators (MKK7 and MK2) of MAPK signaling. We also posit that the junction restructuring event at the apical ES is affected by β-catenin's ability to regulate factors such as Lrrn3 and Vps33a, which are implicated in recycling and degrading proteins through endocytosis [24], [25], [26]. Since β-catenin is known to play an important role in cadherin recycling at the epithelial cell surface [77], we propose that β-catenin deletion would result in altered recycling and degradation of cadherin on the germ cell surface leading to loss of Sertoli cell-germ adhesion at the apical ES. In addition to Sertoli cell-germ cell adhesion, β-catenin may also directly influence vesicle trafficking (and subsequently junction export and acrosome formation) and germ cell polarity by interacting with polarity proteins JAM-C and Cdc42. Finally, β-catenin may regulate chromatin compaction during spermatid development by affecting the expression of Dtl and Arpc5, which we and others have shown to regulate chromatin compaction of differentiating germ cells.

To further understand the mechanism by which β-catenin may regulate differentiation/maturation of post-meiotic germ cells, we performed gene expression analysis on RNA isolated from purified round spermatid as well as whole testis from 6 to 8-week old control and *Ctnnb1 F*Δ mice. Our analysis revealed altered expression of a number of genes encoding proteins involved in cellular movement, tissue morphology, cell signaling, and molecular transport (raw data available at NCBI GEO, accession #GSE30773). One group of genes of particular interest was those involved in the mitogen-activated protein kinase (MAPK) pathway, which has been shown to be involved in cytoskeletal rearrangements [21], [22], [23]. A number of kinase genes in the MAPK pathway, including *Map2k7* (MKK7) and *Mapkapk2* (MK2) were found to be upregulated, while phosphatase genes such as *Dusp26* was downregulated (Table S2 and Figure 6A). Another group of genes with altered expression are those involved in receptor recycling and degradation such as *Lrrn3* and *Vps33a* (Table S2 and Figure 6A) [24], [25], [26]. In addition, the expression of *Dtl*, a member of the E3 ubiquitin ligase family, was altered (Table S2 and Figure 6A). Dtl is involved in the degradation of various cell cycle proteins, including those that regulate chromatin compaction [27], [28], [29]. Finally, β-catenin knockout resulted in increased expression of *Arpc5* (Table S2 and Figure 6A), which we recently showed to play an important role in chromatin compaction by regulating translational activation of post-meiotic germ cell transcripts including protamines (Chang *et al.*, unpublished observations, in submission).

## Discussion

Developing germ cells must be in intimate contact with Sertoli nurse cells for the successful completion of spermatogenesis. This cellular interaction is facilitated by adherens junctional complexes such as ectoplasmic specializations (ES), which is critical for both adhesion and signaling between Sertoli cells and between Sertoli cells and germ cells [7]. The importance of Sertoli cell-germ cell interaction is evidenced by the fact that abnormal or disrupted ES contributes to spermatid sloughing and oligospermia in pathological conditions associated with reduced fertility potential, including varicocele, hyperprolactinemia, diabetes and idiopathic oligospermia [5], [30], [31]. Despite its critical importance, underlying mechanisms that regulate Sertoli cell-germ cell adhesion and signaling remains to be completely understood. Historically, Sertoli cells were believed to be the sole contributor to adhesion and signaling functions required for germ cell differentiation and maturation. Here, we provide evidence that germ cells play an equally important role in Sertoli cell-germ cell adhesion and signaling necessary for the generation of fertilization-competent spermatozoa. Our results revealed that spermatid-specific deletion of β-catenin, which we show to be highly expressed in post-meiotic germ cells (Figure 1B-1D), resulted in significantly reduced sperm count, compromised sperm motility, and impaired fertility in mice (Figure 2C-2H). These defects are due to increased germ cell apoptosis (Figure 3A-3C), defective chromatin compaction (Figure 4A and 4B), disruption of adherens junctions (apical ES), and impaired spermatid polarization observed in our β-catenin-deleted mice (Figure 5A and 5D).

Because β-catenin is also known to be highly expressed in Sertoli cells [6], we reasoned that loss of adherens junction in our β-catenin-deleted mice may be due to disruption of the Sertoli cell β-catenin complex binding to the β-catenin complex on the germ cell surface. Supporting this notion, the interaction of β-catenin-cadherin complexes between adjacent cells is reported to be essential for cell-cell adhesion in epithelial cells [32], [33]. Previous studies have suggested that Sertoli cell-germ cell adherens junctions restructuring (adhesion and dissociation), which is essential for germ cell maturation and spermiation [7], [34], [35], [36], is likely facilitated by signaling and cell adhesion events acting in tandem. We propose that β-catenin may be one such molecule that couples physical adhesion and signaling at Sertoli cell-germ cell interfaces. One mechanism by which β-catenin can accomplish this is by regulating the stability and promoting rapid presentation of cadherin at the ES surface, as it acts as a chauffeur to facilitate the transport of cadherin out of endoplasmic reticulum to the cell surface [32]. In addition to playing a direct role, β-catenin may indirectly regulate the restructuring process by coordinating cross-talk among different signaling pathways.

One example of a β-catenin-regulated pathway may be the MAPK cascade, components of which showed altered expression in β-catenin-deleted mice. Gene expression analyses revealed that β-catenin deletion resulted in elevated expression of *Map2k7* (MKK7) and *Mapkapk2* (MK2) (Table S2 and Figure 6A), which may be directly involved in actin restructuring and adherens junction kinetics by activating downstream JNK and p38 MAPK (Figure 6B) [37], [38], [39], [40], [41], [42], [43], [44], [45], [46]. Furthermore, members of this kinase cascade have been shown to be activated by Axin and Dishevelled [47], [48], factors that regulate β-catenin signaling [49], or by Rho small GTPases Cdc42 and Rac1 [50], [51], [52], [53], which have all been implicated in actin cytoskeleton restructuring (Figure 6B) [54], [55]. In addition, β-catenin knockdown reduced the expression of *Dusp26*, a member of the dual specificity protein phosphatase family known to negatively regulate p38 MAPK (Table S2 and Figure 6A) [56], [57], [58]. Together, these results suggest that β-catenin stabilizes the actin cytoskeleton network on germ cell surfaces by activating negative regulators and inhibiting positive regulators of MAPK signaling (Figure 6B).

Another group of genes that showed altered expression in β-catenin-deleted germ cells are those that mediate receptor recycling and degradation through endocytosis. Several genes involved in these processes, such as *Vps33a* and *Lrrn3*, were found to be downregulated in β-catenin-deleted testes (Table S2 and Figure 6A). Vps33a is involved in recruitment of endosomes and multivesicular bodies to lysosomes [24], [25], while Lrrn3 is believed to facilitate the internalization of EGFR [26], a known binding partner of cadherin [59]. Since adherens junctions between Sertoli cell and germ cell must undergo extensive restructuring to facilitate germ cell maturation [7], it is economical not to synthesize proteins *de novo* each time junctional restructuring takes place. Instead, rapid recycling of the signaling proteins is likely to coordinate the restructuring processes. Therefore, it is possible that the reduced expression of *Vps33a* and *Lrrn3* in β-catenin-deleted mice may impair the process of β-catenin-mediated recycling of cadherin on the germ cell resulting in loss of Sertoli cell-germ adhesion at the apical ES (Figure 6B).

Our results reveal that in addition to adhesion, β-catenin may play a critical role in spermatid polarization. Since JAM-C colocalized with β-catenin on the on the distal side of germ cells (Figure 5B), the side which is in constant contact with Sertoli cells [9], it is likely that β-catenin-JAM-C-Cdc42-mediated assembly of spermatid polarization complex is directly associated with Sertoli cell-germ cell adhesion (Figure 6B). Consistent with this, cell-cell adhesion is reported to induce epithelial cell polarity via cadherin and β-catenin [60], [61]. Moreover, the loss of Par6, which is a polarity complex protein, is associated with destabilization of actin filament at the apical ES and the loss of adhesion function [62]. Future studies aimed at understanding the role of β-catenin target genes will provide greater insight into the mechanism by which β-catenin regulates spermatid polarization events.

The apical ES is first formed between Sertoli cells and round/elongate spermatids at step 8 of the seminiferous epithelial cycle and stays throughout the epithelial cycle until step 16 in mice [9], a stage when most morphological changes including elongation of round spermatid nuclei and compaction of their chromatin occur. Our results demonstrating defective chromatin condensation in differentiating germ cells of β-catenin-deleted mice suggest a specialized role for β-catenin in this process (Figure 4A and 4B). One molecule that may play a crucial role in this process is actin-associated protein Arpc5 that shows induced expression in our β-catenin-deleted mice (Table S2 and Figure 6A) [63], [64], [65]. We have recently shown that Arpc5 regulates chromatin compaction event by controlling the distribution of germ cell mRNAs, including protamines, between translationally active and inactive pool (Chang et al., unpublished observations, submitted). Another gene that may be associated with altered chromosome compaction in β-catenin-deleted mice is *Dtl* (Table S2 and Figure 6A), a member of the CRL4 E3 ubiquitin ligase complex [27] that prevents premature chromatin condensation during S phase [28].

Our results show that β-catenin deletion in spermatids resulted in increased apoptosis of both post-meiotic and pre-meiotic germ cells (Figure 3A-3C). While apoptosis of post-meiotic germ cells is conceivable, it is intriguing that pre-meiotic germ cells also underwent increased cell death in our β-catenin-deleted mice. One plausible explanation could be that there is a bi-directional cross talk between basal ES/tight junction at the blood-testis barrier and apical ES; such that disruption of apical ES due to β-catenin deletion may lead to perturbed signaling at the basal ES/tight junction, resulting in impaired pre-meiotic germ cell development and apoptosis. In agreement with this, recent studies have shown that disruption of Sertoli cell-spermatid adhesion at the apical ES due to blockage of laminin-333 activity results in impaired functioning of basal ES/tight junction at the blood-testis barrier [66]. Moreover, junctional protein complexes at the basal ES such as catenin-cadherin, nectin-afidin and integrin-laminin are also present at the apical ES [67], [68], [69], further supporting the idea that junctional restructuring events at one end must be affecting restructuring events at the other end of the Sertoli cell.

In conclusion, our findings suggest that β-catenin is a key molecule that couples cell adhesion with signaling events to ensure proper germ cell differentiation. Our studies reveal that β-catenin in post-meiotic germ cells plays an important role in wide array of specific events during germ cell maturation including Sertoli cell-germ cell adhesion at the apical ES, spermatid polarization and chromatin condensation. Future studies will shed lights on the precise mechanism by which β-catenin target genes mediate Sertoli cell-germ adhesion at the apical ES and germ cell differentiation events.

## Materials and Methods

### Animals, genotyping, and reproductive phenotype analyses

All animal experiments were performed in accordance with the National Institutes of Health Guide for the Care and Use of Laboratory Animals. Approval of animal use for this study was granted by The Institutional Animal Care and Use Committee of The University of Texas Health Science Center at San Antonio (Animal Welfare Assurance #A3345-01; Protocol #07057-34-02-A). *Prm1-cre* and β*-catenin-flox* mice were obtained from Jackson Laboratories [12], [13], housed in a barrier facility, and placed on 12 h light and 12 h dark cycles. Primers used for genotyping are previously described [16], [70]. To obtain sperm count and motility, 6 to 8-week old male mice were euthanized by CO_2_, and caudal epididymides were harvested in modified Krebs-Ringer (mKR) medium. Small cuts were made in the epididymides and sperm were allowed to disperse into the medium for 15 min at 37°C. Sperm was diluted 1∶10 before counting on a hemocytometer. For motility, an aliquot of sperm in mKR medium was loaded into a pre-warmed counting chamber and examined in triplicate. Sperm were scored to be progressively motile or non-motile as previously described [71]. Tissue preparation, eight-week timed matings, sonication-resistant spermatid count, hematoxylin and eosin (HE) staining, and TUNEL assay were conducted as previously described [72]. Progeny from matings were genotyped to determine efficiency of cre-loxP recombination in round spermatids.

### Elutriation, RNA analyses, and protein analyses

Testes harvested from four 6 to 8-week old mice were elutriated as previously described [11], with modifications. Briefly, total testicular single cell suspensions produced from enzymatic digests were separated by centrifugal elutriation on the JE-5.0 rotor (Beckman Coulter) to obtain fractions enriched in elongating spermatids (12 ml/min, 2,000 rpm), round spermatids (15 ml/min, 2,000 rpm), pachytene spermatocytes (30 ml/min, 2,250 rpm), and Sertoli cells (65 ml/min, stop rotor). Further purification of round spermatids and pachytene spermatocytes were performed by ultracentrifugation through 28-45% and 26-38% Percoll (GE Healthcare) gradient at 10,000 rpm, respectively. Further purification of Sertoli cells were performed by allowing cells to adhere to datura-coated plates followed by hypotonic shock with 0.3x HBSS for 3 min. The purity of the preparations were determined (∼90%) by light microscopic examination of periodic acid Schiff (PAS)/hematoxylin-stained spermatogenic cells (PAS Staining System, Sigma) and qPCR analyses using primers for *Ctnnb1*, *Sycp3* (pachytene spermatocytes), *Acrv1* (round spermatids), *Prm1* (round/elongating/elongated spermatids), *Dbil5* (elongating/elongated spermatids), *Gata1* (Sertoli cells), and *Rhox5* (Sertoli cells). Enriched cells were processed to isolate total RNA using Trizol (Invitrogen). cDNA was synthesized with iScript cDNA Synthesis Kit (Bio-rad) and analyzed by qPCR using iQ SYBR Green Supermix (Bio-rad). Expression levels were normalized to mouse *Rpl19*.

### Immunofluorescence and immunohistochemistry

Enriched spermatid populations from elutriation or caudal epididymal spermatozoa were air dried on slides, fixed with 4% paraformaldehyde, and permeabilized with cold methanol at -20°C for 10 min. Paraffin-embedded testis sections were rehydrated and antigen retrieval was performed by boiling in 10 mM sodium citrate for 30 min. Samples were then blocked with 10% goat serum, and incubated with primary antibodies in 3% goat serum overnight at 4°C. Samples were incubated with secondary antibodies in 3% goat serum for 1 h and mounted with Vectashield Hard Set Mounting Medium with DAPI (Vector Laboratories). For immunohistochemistry, sections were developed with 3,3′-diaminobenzidine (DAB; Sigma) and counterstained with Mayer's hematoxylin (Sigma) after incubation with secondary antibody. Slides were then dehydrated and mounted with Cytoseal XYL (Richard-Allan). Antibodies and concentrations used are described in figure legends. All photomicrographs were taken on the Nikon Eclipse TE2000-U.

### Nuclear cytoplasmic protein fractionation

Isolation of nuclear and cytoplasmic fractions from germ cells was performed as previously described [73]. Briefly, purified germ cells from elutriation were lysed in NP40 solution [10 mM Tris-HCl, pH 8.0|0.1 mM EDTA, pH 8.0|150 mM NaCl|0.6% NP40|0.04 mM PMSF|0.04% Protease Inhibitor Cocktail (Sigma)], mixed by pipetting, and incubated on ice for 10 min. After centrifugation for 2 min at 4,200 rpm the supernatant was saved as the cytoplasmic fraction. The nuclear pellet was washed in DOC solution [10 mM Tris-HCl, pH 8.0|0.1 mM EDTA, pH 8.0|150 mM NaCl|0.6% NP40|0.5% sodium deoxycholate|0.04 mM PMSF|0.04% Protease Inhibitor Cocktail (Sigma)] and mixed by pipetting. After centrifugation for 2 min at 7,500 rpm the supernatant was discarded, and the nuclear pellet was lysed in CellLytic M Cell Lysis Reagent (Sigma) with 0.04 mM PMSF and 0.04% Protease Inhibitor Cocktail. Western blot analyses were performed as previously described with antibodies described in figure legends [72].

### Spermatozoa staining and flow cytometry

Acridine orange staining of caudal spermatozoa and subsequent analyses by flow cytometry were performed as previously described [74].

### Electron microscopy

Electron microscopy was performed as previously described [72], [75].

### Co-immunoprecipitation (co-IP)

Total testis lysate was prepared by Dounce homogenization in 1 ml IP lysis buffer [50 mM Tris-HCl, pH 8.0|50 mM EDTA, pH 8.0|150 mM NaCl|0.5% NP40|1 mM DTT|1% Protease Inhibitor Cocktail]. Co-IP was carried out using Dynabeads Protein G Immunoprecipitation Kit (Invitrogen) according to manufacturer protocols. Briefly, 1.5 mg of protein lysate was precleared with 50 µl Dynabeads for 1 h. 5 µg of anti-β-catenin (Sigma) or normal rabbit IgG (Santa Cruz) were incubated with 50 µl Dynabeads for 30 min with rotation. Pre-cleared lysates were incubated with antibody-conjugated beads overnight at 4°C with rotation. Beads were collected, washed twice, and bound proteins were eluted by boiling in Laemmli sample buffer. Protein was visualized by western blot analyses using antibodies described in figure legends.

### Microarray

Total RNA from purified round spermatids (pooled from four animals for both control and *Ctnnb1 F*Δ mice) and whole testis (from two control and two *Ctnnb1 F*Δ mice) were hybridized to the Agilent 4×44 k Whole Mouse Genome Microarray according to manufacturer's protocol and scanned on the Agilent G2505B scanner. Expression levels of selected genes were further verified by qPCR analyses on RNA extracted from purified round spermatids. We have deposited the raw data at National Center for Biotechnology Information Gene Expression Omnibus (accession #GSE30773), and confirm that all details are MIAME compliant.

### Statistical analysis

All values and error bars in graphs are means ± SEM; respective *n* values are indicated in figure legends; *p*-values are determined by two-tailed Student's *t*-tests.

## Supporting Information

Figure S1 **Loss of** **β-catenin expression in** ***Ctnnb1 F***Δ **post-meiotic germ cells.**Testis sections from control and *Ctnnb1 F*Δ mice were labeled with anti-β-catenin primary antibody (1∶200) and HRP-conjugated goat anti-rabbit secondary antibody (1∶800; Santa Cruz). Sections were developed with DAB and counterstained with Mayer's hematoxylin. While round and elongating spermatids clearly showed β-catenin expression in control seminiferous tubules (green and white arrowheads, respectively; panel c), no detectable β-catenin staining was observed in the round and elongating spermatids of *Ctnnb1 F*Δ tubules (blue and yellow arrowheads, respectively; panel f), suggesting that both copies of the β*-catenin-flox* allele has been conditionally deleted. Please note that elongating spermatids exhibited only purple hematoxylin staining in *Ctnnb1 F*Δ tubules (panels d-f), suggesting no β-catenin expression, while elongating spermatids in control tubules exhibited brown DAB staining (panels a-c). β-catenin staining in Sertoli cells remained unchanged in *Ctnnb1 F*Δ compared to control tubules (arrows; panels c and f). Areas in black boxes are magnified (panel a in panel b; panel d in panel e), and areas in red boxes are further magnified (panel b in panel c; panel e in panel f). Scale bar, 100 µm (panels a and d), 50 µm (panels b and e), or 25 µm (panels c and f).
(TIF)

Figure S2 **Loss of** **β-catenin expression in** ***Ctnnb1 F***Δ **elongating spermatids.** Testis sections from control and *Ctnnb1 F*Δ mice were labeled with anti-β-catenin (1∶50) followed by AlexaFluor 488-conjugated goat anti-rabbit (1∶400). Sections were counterstained with DAPI (blue) for nuclear staining. While elongating spermatids clearly showed β-catenin expression in control seminiferous tubules (white arrowheads; panel d), no detectable β-catenin staining was observed in the elongating spermatids of *Ctnnb1 F*Δ tubules (yellow arrowheads; panel h). β-catenin staining in Sertoli cells remained unchanged in *Ctnnb1 F*Δ compared to control tubules (arrows; panels d and h). Areas in boxes are magnified (panel c in panel d; panel g in panel h). Scale bar, 50 µm (panels a-c and e-g) or 25 µm (panels d and h).
(TIF)

Figure S3 ***Prm1-cre*** **males exhibit no reproductive defect.** (**A**) Mean number of litters (*n* = 10) and (**B**) mean number of pups per litter (*n* = 10) obtained from eight-week timed matings of 6 to 8-week old *Prm1-cre* and control littermates.
(TIF)

Figure S4 **Reduction in** ***Ctnnb1 F***Δ **testis size.** Testis from a control (left) and a *Ctnnb1 F*Δ (right) mouse, showing a modest reduction of testis size when *β-catenin* is conditionally deleted in haploid spermatids.
(TIF)

Table S1 ***β-catenin*** **expression in enriched Sertoli and germ cell populations.**QPCR analyses of RNA from purified Sertoli and spermatogenic cell populations pooled from four mice using primers in Table S3. PS, pachytene spermatocyte; RS, round spermatid; ES, elongating/elongated spermatid.
(DOC)

Table S2 **List of highly altered genes in** ***Ctnnb1 F***Δ **post-meiotic germ cells. **Genes listed were found to be highly altered (upregulated or downregulated) in total testis and purified round spermatids of *Ctnnb1 F*Δ mice compared to control mice. A complete list of altered genes in *Ctnnb1 F*Δ total testis and purified round spermatids microarrays is available at NCBI GEO (accession #GSE30773).
(DOC)

Table S3 **Primers used in this study.** Primer sequences were obtained from PrimerBank [78].
(DOC)
