# Supplementary figures and images for: Role of β-Catenin in Post-Meiotic Male Germ Cell Differentiation
Source: PLoS One. 2011 Nov 18;6(11):e28039. doi: 10.1371/journal.pone.0028039 (PMC3220672; doi:10.1371/journal.pone.0028039)

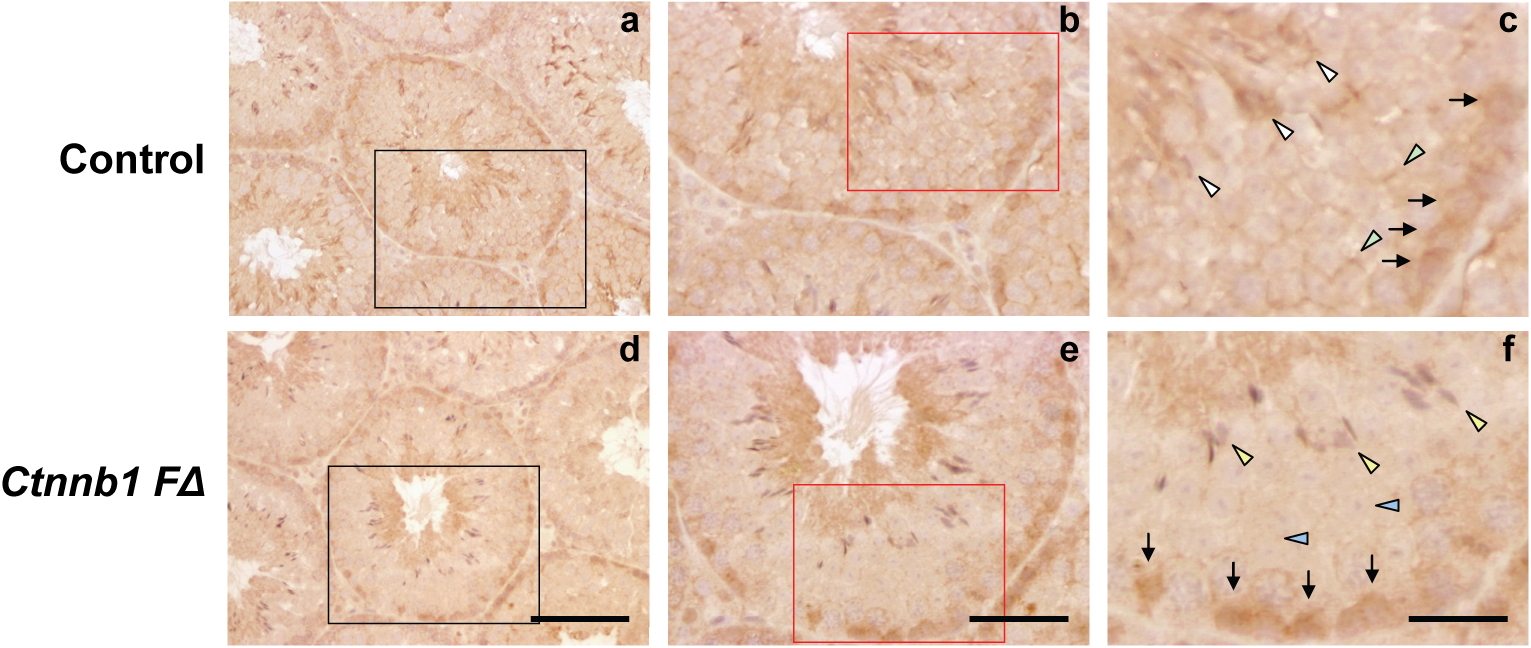

Supplement: Figure S1 — Loss of β-catenin expression in Ctnnb1 FΔ post-meiotic germ cells.Testis sections from control and Ctnnb1 FΔ mice were labeled with anti-β-catenin primary antibody (1∶200) and HRP-conjugated goat anti-rabbit secondary antibody (1∶800; Santa Cruz). Sections were developed with DAB and counterstained with Mayer's hematoxylin. While round and elongating spermatids clearly showed β-catenin expression in control seminiferous tubules (green and white arrowheads, respectively; panel c), no detectable β-catenin staining was observed in the round and elongating spermatids of Ctnnb1 FΔ tubules (blue and yellow arrowheads, respectively; panel f), suggesting that both copies of the β-catenin-flox allele has been conditionally deleted. Please note that elongating spermatids exhibited only purple hematoxylin staining in Ctnnb1 FΔ tubules (panels d-f), suggesting no β-catenin expression, while elongating spermatids in control tubules exhibited brown DAB staining (panels a-c). β-catenin staining in Sertoli cells remained unchanged in Ctnnb1 FΔ compared to control tubules (arrows; panels c and f). Areas in black boxes are magnified (panel a in panel b; panel d in panel e), and areas in red boxes are further magnified (panel b in panel c; panel e in panel f). Scale bar, 100 µm (panels a and d), 50 µm (panels b and e), or 25 µm (panels c and f). (TIF) [file pone.0028039.s001.tif]

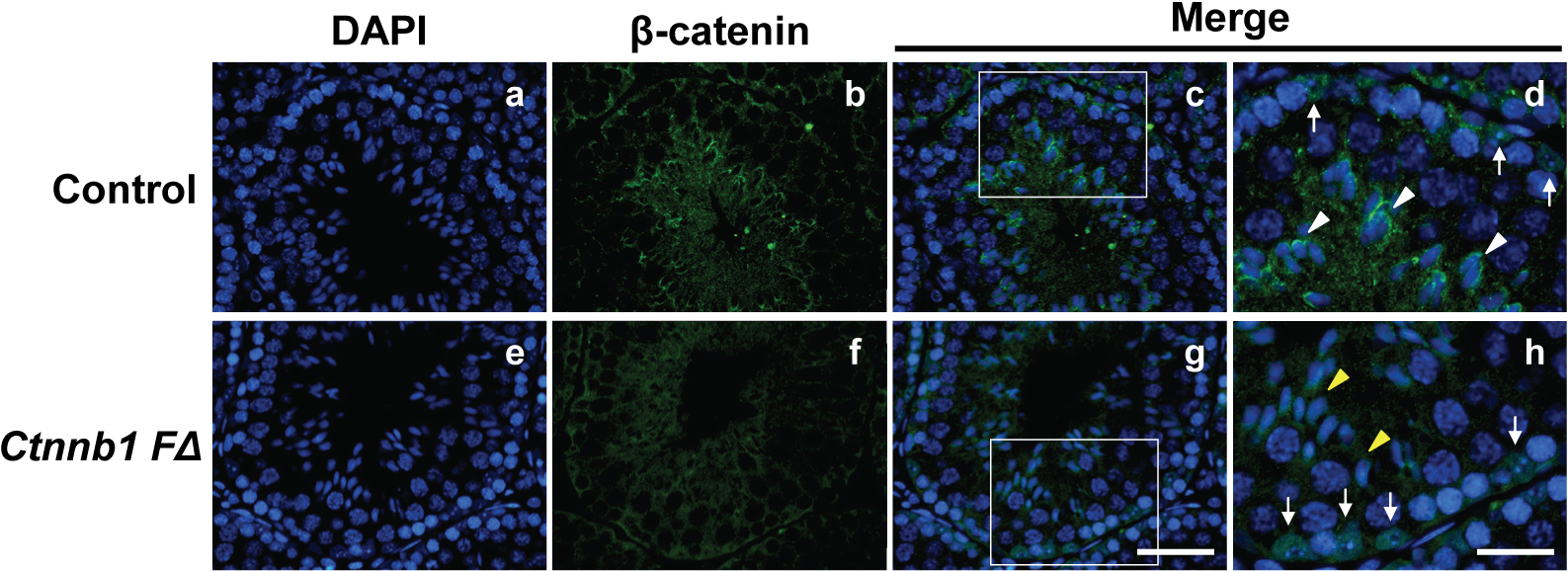

Supplement: Figure S2 — Loss of β-catenin expression in Ctnnb1 FΔ elongating spermatids. Testis sections from control and Ctnnb1 FΔ mice were labeled with anti-β-catenin (1∶50) followed by AlexaFluor 488-conjugated goat anti-rabbit (1∶400). Sections were counterstained with DAPI (blue) for nuclear staining. While elongating spermatids clearly showed β-catenin expression in control seminiferous tubules (white arrowheads; panel d), no detectable β-catenin staining was observed in the elongating spermatids of Ctnnb1 FΔ tubules (yellow arrowheads; panel h). β-catenin staining in Sertoli cells remained unchanged in Ctnnb1 FΔ compared to control tubules (arrows; panels d and h). Areas in boxes are magnified (panel c in panel d; panel g in panel h). Scale bar, 50 µm (panels a-c and e-g) or 25 µm (panels d and h). (TIF) [file pone.0028039.s002.tif]

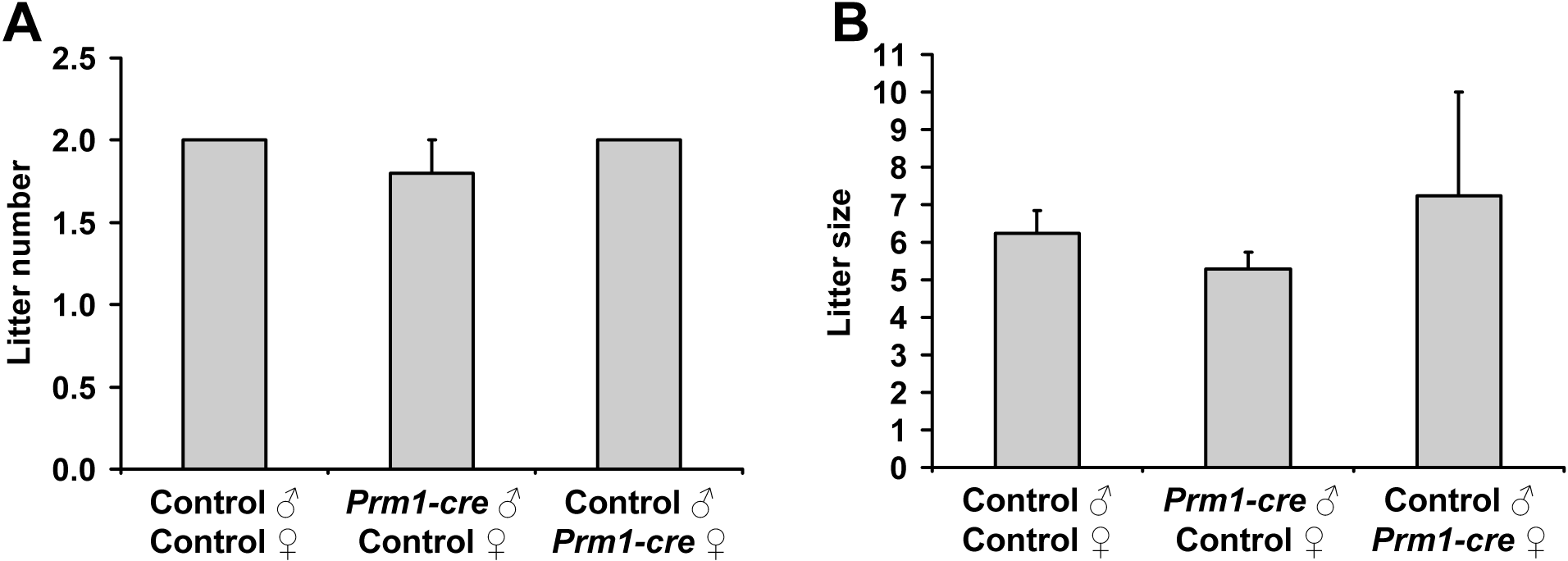

Supplement: Figure S3 — Prm1-cre males exhibit no reproductive defect. (A) Mean number of litters (n = 10) and (B) mean number of pups per litter (n = 10) obtained from eight-week timed matings of 6 to 8-week old Prm1-cre and control littermates. (TIF) [file pone.0028039.s003.tif]

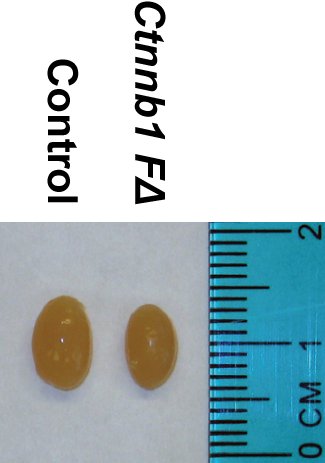

Supplement: Figure S4 — Reduction in Ctnnb1 FΔ testis size. Testis from a control (left) and a Ctnnb1 FΔ (right) mouse, showing a modest reduction of testis size when β-catenin is conditionally deleted in haploid spermatids. (TIF) [file pone.0028039.s004.tif]
