# Supplementary material for: Role of β-Catenin in Post-Meiotic Male Germ Cell Differentiation
Source: PLoS One. 2011 Nov 18;6(11):e28039. doi: 10.1371/journal.pone.0028039 (PMC3220672; doi:10.1371/journal.pone.0028039)
Supplement: Table S1 — β-catenin expression in enriched Sertoli and germ cell populations.QPCR analyses of RNA from purified Sertoli and spermatogenic cell populations pooled from four mice using primers in Table S3. PS, pachytene spermatocyte; RS, round spermatid; ES, elongating/elongated spermatid. (DOC) [file pone.0028039.s005.doc]

**Table S1. *β-catenin* expression in enriched Sertoli and germ cell populations.**

| **Gene** | **Sertoli Cell** | **Germ Cell** | | | **Primary Expression** |
| --- | --- | --- | --- | --- | --- |
|  |  | **PS** | **RS** | **ES** |  |
| ***Ctnnb1*** | 4.73 | 2.80 | 2.72 | 2.30 | Sertoli/Germ |
| ***Dbil5*** | 0.30 | 0.50 | 1.10 | 4.85 | ES |
| ***Prm1*** | 0.15 | 0.24 | 2.68 | 5.20 | RS/ES |
| ***Acrv1*** | 0.63 | 0.54 | 5.80 | 0.65 | RS |
| ***Sycp3*** | 0.58 | 3.60 | 0.05 | 0.10 | PS |
| ***Gata1*** | 4.60 | 0.36 | 0.26 | 0.18 | Sertoli |
| ***Rhox5*** | 3.80 | 0.25 | 0.28 | 0.32 | Sertoli |

QPCR analyses of RNA from purified Sertoli and spermatogenic cell populations pooled from four mice using primers in Table S3. PS, pachytene spermatocyte; RS, round spermatid; ES, elongating/elongated spermatid.
