# Supplementary material for: Role of β-Catenin in Post-Meiotic Male Germ Cell Differentiation
Source: PLoS One. 2011 Nov 18;6(11):e28039. doi: 10.1371/journal.pone.0028039 (PMC3220672; doi:10.1371/journal.pone.0028039)
Supplement: Table S2 — List of highly altered genes in Ctnnb1 FΔ post-meiotic germ cells. Genes listed were found to be highly altered (upregulated or downregulated) in total testis and purified round spermatids of Ctnnb1 FΔ mice compared to control mice. A complete list of altered genes in Ctnnb1 FΔ total testis and purified round spermatids microarrays is available at NCBI GEO (accession #GSE30773). (DOC) [file pone.0028039.s006.doc]

**Table S2. List of highly altered genes in *Ctnnb1 FΔ* post-meiotic germ cells.**

| **Gene** | **Fold Change** | **Function and Expression** | **References** |
| --- | --- | --- | --- |
| ***Arpc5*** | 2.69 | subunit of the Arp2/3 complex involved in vesicle trafficking and cell polarity by stimulating actin filament assembly | [63,64,65] |
| ***Dtl*** | -3.02 | member of the CRL4 E3 ubiquitin ligase complex; prevents premature chromatin compaction in S phase of cell cycle; CRL4 complex is important in male fertility | [27,28,29] |
| ***Dusp26*** | -3.59 | inhibits p38 MAPK; facilitates transport of β-catenin/cadherin complex to the cell surface by associating with and dephosphorylating microtubule-associated motor proteins | [56,57,58] |
| ***Lrrn3*** | -3.09 | possible role of mediating endocytosis of EGFR; enhances MAPK activation by EGF | [26] |
| ***Map2k7*** | 7.03 | activates JNK; downstream target of Axin, Dishevelled, Cdc42, and Rac1; testis-restricted expression of an alternatively spliced transcript | [37,38,48,52] |
| ***Mapkapk2*** | 3.18 | activated by p38 MAPK; phosphorylates Arpc5; involved in cell migration through restructuring of the actin cytoskeleton | [40,41,42,43,46] |
| ***Vps33a*** | -2.11 | facilitates fusion of endosomes to lysosomes in Drosophila; facilitates recruitment of multivesicular bodies to lysosomes to degrade apical surface protein complexes in mouse bladder cells | [24,25] |

Genes listed were found to be highly altered (upregulated or downregulated) in total testis and purified round spermatids of *Ctnnb1 FΔ* mice compared to control mice. A complete list of altered genes in *Ctnnb1 FΔ* total testis and purified round spermatids microarrays is available at NCBI GEO (accession #GSE30773).
