# Supplementary material for: Role of β-Catenin in Post-Meiotic Male Germ Cell Differentiation
Source: PLoS One. 2011 Nov 18;6(11):e28039. doi: 10.1371/journal.pone.0028039 (PMC3220672; doi:10.1371/journal.pone.0028039)
Supplement: Table S3 — Primers used in this study. Primer sequences were obtained from PrimerBank [78]. (DOC) [file pone.0028039.s007.doc]

**Table S3. Primers used in this study.**

| **Primer** | **Sequence** |
| --- | --- |
| ***Acrv1*** | 5’-TCAGCAACTTTCAAGCGAGTAT-3’  5’-CTCCTGAAGAGTGCTCACCTG-3’ |
| ***Arpc5*** | 5’-GTGCAGGCAGCATCGTCTT-3’  5’-CATTAGGAGGTCCACACCGTT-3’ |
| ***Dbil5*** | 5’-CCCAGGGCGACTGTAACATC-3’  5’-GCAATGTAGATCCTCATGGCAT-3’ |
| ***Dtl*** | 5’-ACGCAAGGCAGAAAATTCATCC-3’  5’-GAGCTGGGAGTAATGGTGACC-3’ |
| ***Dusp26*** | 5’-AACGCCTCACACAACAGGTG-3’  5’-AGCCGTCTGAAAGTGGATGC-3’ |
| ***Gata1*** | 5’-ACTGGCCTACTACAGAGAAGC-3’  5’-GTAGAGTGCCGTCTTGCCATA-3’ |
| ***Lrrn3*** | 5’-ACGTTAATCACAACCTGCTCTC-3’  5’-TGTCCCCAAGCATCAGAATCT-3’ |
| ***Map2k7*** | 5’-ATGGAGAGCATCGAGATTGACC-3’  5’-CGCCGCATTTGCTTAACAG-3’ |
| ***Mapkapk2*** | 5’-TTCCCCCAGTTCCACGTCA-3’  5’-GCAGCACCTTCCCGTTGAT-3’ |
| ***Prm1*** | 5’-CCGTCGCAGGCGAAGATGTC-3’  5’-CACCTTATGGTGTATGAGCGG-3’ |
| ***Rhox5*** | 5’-CACCAGGACCAAAGTGGCC-3’  5’-GGTATGGAAGCTGAGGGTT-3’ |
| ***Rpl19*** | 5’-CTGAAGGTCAAAGGGAATGTG-3’  5’-GGACAGAGTCTTGATGATCTC-3’ |
| ***Sycp3*** | 5’-AGCCAGTAACCAGAAAATTGAGC-3’  5’-CCACTGCTGCAACACATTCATA-3’ |
| ***Vps33a*** | 5’-TACGGGCGAGTGAACCTGAA-3’  5’-ACTCATCCCACACTATTGCCTT-3’ |

Primer sequences were obtained from PrimerBank [78].
